# Mechanistic Aspects of Microbe-Mediated Nanoparticle Synthesis

**DOI:** 10.3389/fmicb.2021.638068

**Published:** 2021-05-05

**Authors:** Shubhrima Ghosh, Razi Ahmad, Kamalika Banerjee, Mohamed Fahad AlAjmi, Shakilur Rahman

**Affiliations:** ^1^Department of Chemistry, Indian Institute of Technology Delhi, New Delhi, India; ^2^Research and Development Office, Ashoka University, Sonepat, India; ^3^Department of Biosciences, Jamia Millia Islamia, New Delhi, India; ^4^Kusuma School of Biological Sciences, Indian Institute of Technology Delhi, New Delhi, India; ^5^Department of Pharmacognosy, College of Pharmacy, King Saud University, Riyadh, Saudi Arabia

**Keywords:** nanoparticles, synthesis mechanism, microbes, metal nanotization, biomaterials, therapeutic

## Abstract

In recent times, nanoparticles (NPs) have found increasing interest owing to their size, large surface areas, distinctive structures, and unique properties, making them suitable for various industrial and biomedical applications. Biogenic synthesis of NPs using microbes is a recent trend and a greener approach than physical and chemical methods of synthesis, which demand higher costs, greater energy consumption, and complex reaction conditions and ensue hazardous environmental impact. Several microorganisms are known to trap metals *in situ* and convert them into elemental NPs forms. They are found to accumulate inside and outside of the cell as well as in the periplasmic space. Despite the toxicity of NPs, the driving factor for the production of NPs inside microorganisms remains unelucidated. Several reports suggest that nanotization is a way of stress response and biodefense mechanism for the microbe, which involves metal excretion/accumulation across membranes, enzymatic action, efflux pump systems, binding at peptides, and precipitation. Moreover, genes also play an important role for microbial nanoparticle biosynthesis. The resistance of microbial cells to metal ions during inward and outward transportation leads to precipitation. Accordingly, it becomes pertinent to understand the interaction of the metal ions with proteins, DNA, organelles, membranes, and their subsequent cellular uptake. The elucidation of the mechanism also allows us to control the shape, size, and monodispersity of the NPs to develop large-scale production according to the required application. This article reviews different means in microbial synthesis of NPs focusing on understanding the cellular, biochemical, and molecular mechanisms of nanotization of metals.

## Introduction

Nanoparticles (NPs) are particles with dimensions between 1 and 100 nm that may have different chemical and physical properties relative to their bulk-metal counterpart in addition to the large surface-to-mass ratio (Sardar et al., 2014). The physical and chemical properties of NPs can be differing by their elemental composition, larger specific surface area, and composition of the coating agents (Ahmad et al., 2013a; Sadaf et al., 2020). NPs possess unique features such as mechanical properties (Mishra et al., 2015; Gao et al., 2020), antimicrobial capabilities (Ahmad et al., 2014a, 2015; Abdulla et al., 2021), drug delivery capacities (Ahmad et al., 2021), optical properties (Albrecht et al., 2018), and catalytic capabilities (Mishra et al., 2016; Liu and Liu, 2017; Perwez et al., 2017; Ghosh et al., 2018a, b) and act as artificial chaperones (Ahmad et al., 2014b; Ghosh et al., 2019).

There are various approaches to synthesize nanomaterials, namely, chemical, physical, and biological methods. In general, the chemical and physical methods are preferred routes by the industries, but their disadvantages overshadow their performance. They are usually expensive, consume enormous amounts of time and energy, have complicated procedures, and generate toxic by-products (Castro et al., 2014; Ngoepe et al., 2020). Biogenic NPs are therefore an alternative with the potential to be an eco-friendly and cost-effective method for NP synthesis (Mishra et al., 2013a; Khatoon et al., 2015; Mazumder et al., 2016; Kim et al., 2018; Jacob et al., 2020). In relation to the manufacture of NPs, the word “biogenic” encompasses a range of methodology used for the NP synthesis by either plant extracts or microbes by reduction of metal ions into NPs through their inherent NP manufacturing capabilities (Capeness et al., 2019; Castillo-Henriquez et al., 2020). Bacterial growth properties and genetic modifications render them potent for biogenic NP manufacture as well as industrial applications, particularly when combined with their ability to catch metals, such as those found as environmental pollutants (Nancharaiah et al., 2016).

Interactions between inorganic matter and biological entities have been responsible for geochemical cycles and the maintenance of life on this planet for millions of years. Many species during evolution rely on minerals of different sizes and shapes for a broad range of functions, including physical support, defense from foreign agents, and navigation (Wang and Nilsen-Hamilton, 2013). Biomineralization requires the absorption and modulation of ions from the atmosphere into highly ordered structures, activities that are subject to strict biological regulation (Mann, 2001). A solid-phase organic matrix comprising polysaccharides, phospholipids, and mostly proteins is needed to generate higher-order inorganic structures (Addadi and Weiner, 1985, 2014; Weiner and Dove, 2003). The processes of inorganic structure formation, nucleation, crystal growth, and developing minerals of particular size as well as shape are involved. Harsh conditions may be required to form minerals in the environment. To overcome these situations, organisms provide an offshore account in which specific biological systems help with specific membrane pumps to produce a saturating level of a specific ion (Weiner and Dove, 2003). During mineral formation, the organic matrix plays a major role. First of all, negatively charged residues pull and concentrate positive ions from solution. This contributes to ion saturation at particular points to initiate the nucleation. Nucleation involves a reduction in the free energy and formation of nano-size particles. As a result, the organic matrix decreases the free energy and stabilizes the ions to create a solid stable particle that will be growing into a crystal. Also, the organic matrix plays an important role in the crystal growth and formation of definite particle size. Earlier research has shown that diverse proteins can directly interact with the mineral surface for the formation of solid structure (Hunter, 1996; Mann, 2001; Addadi and Weiner, 2014; Nudelman et al., 2018). A specific organelle called magnetosome, which contains nano-magnetic particles synthesized by magnetotactic bacteria (MTB), is a good model to understand NP formation (Lower and Bazylinski, 2013). The magnetosome is composed of well-defined nano-magnetic particles in a chain or chains that are enclosed by a membrane layer and oriented as per the cell axis (Yamamoto et al., 2010). This magnetosome is often exploited to carry drugs or other loads as a delivery vehicle in cancer treatments (Vargas et al., 2018).

As is evident, several metals such as gold, silver, copper, cadmium, zinc, tellurium, platinum, titanium, and palladium are found to be synthesized through microbial routes, which are often harvested for industrial or biomedical applications. This mini review covers the microbial route to synthesize the various nanomaterials, focusing on the mechanistic aspects of nanotization.

## Microbial-Mediated Synthesis of Nanoparticles

**FIGURE 1:**
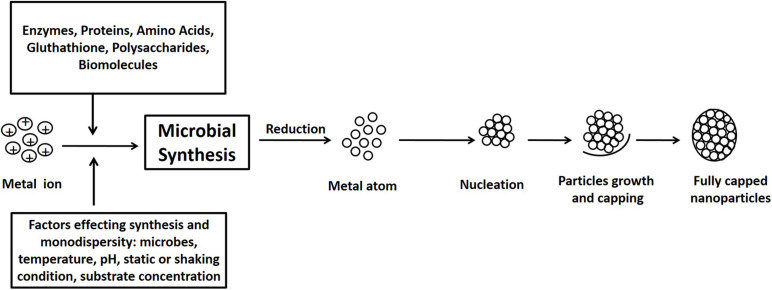
Schematic diagram of mechanisms of nanoparticle synthesis by microbes: the pathway of nanoparticle synthesis by microbes involves metal capture, enzymatic reduction, and capping. Several biomolecules such as proteins, amino acids, and polysaccharides found in the microbial extracts help in the stabilization of the nanoparticles.

Microorganisms such as bacteria, fungi, yeast, and algae are often favored for NP synthesis due to simpler cultivation, rapid growth rate, and their capacity to grow at atmospheric pH, temperature, and pressure conditions. Different biological agents behave differently with different metal solutions in order to form NPs (Fariq et al., 2017; Saravanan et al., 2020; Ghosh et al., 2021). Metal ions are initially trapped on the surface of the cell followed by the reduction of metal ions to NPs with the presence of enzymes synthesized by the microbes. The NP synthesis process is depicted in Figure 1. The microbial-mediated synthesis of NPs and their possible application is summarized in Table 1.

**TABLE 1:** Nanoparticles synthesized by microbe and their possible application.

Microbial strain	Nanoparticles	Size in nm	Application	References
*Micrococcus lylae*	TiO_2_	13.58 ± 0.04	Degradation of dye	Fulekar et al., 2018
*Cellulosimicrobium* sp.	TiO_2_	15.76 ± 0.03	Degradation of dye	Fulekar et al., 2018
*Micrococcus aloeverae*	TiO_2_	17.31 ± 0.02	Degradation of dye	Fulekar et al., 2018
*Chlorella pyrenoidosa*	TiO_2_	50	Dye degradation	Sharma et al., 2018
*Bacillus mycoides*	TiO_2_	40–60	Construction of green solar cells	Ordenes-Aenishanslins et al., 2014
*Lactobacillus* sp.	TiO_2_	50–100	Antibacterial activity, immobilization, and refolding of enzyme	Ahmad et al., 2013a, 2014a
*Aspergillus flavus*	TiO_2_	62–74	Antimicrobial activity	Rajakumar et al., 2012
*Marinospirillum alkaliphilum*	Ag	30–70	Antimicrobial effect and dye removal	Nazari and Kashi, 2021
*Escherichia coli*	Ag	5–50	Antimicrobial activity	Saeed et al., 2020
*Pseudoduganella eburnean*	Ag	8–24	Antimicrobial activity	Huq, 2020
*Sphingobium* sp. MAH-11^T^	Ag	7–22	Antibacterial activity	Akter and Huq, 2020
*Bacillus subtilis*	Ag	3–20	Antibacterial activity	Alsamhary, 2020
*Lactobacillus plantarum* TA4	Ag	14.0 ± 4.7	Antibacterial and Antioxidant activity	Mohd Yusof et al., 2020
*Padina* sp.	Ag	25–60	Antibacterial activity	Bhuyar et al., 2020
*Chaetomorpha linum*	Ag	70–80	Efficient anticancer agent	Acharya et al., 2020
*Chlorella ellipsoidea*	Ag	220.8 ± 31.3	Photophysical, catalytic, and antibacterial activity	Borah et al., 2020
*Penicillium oxalicum*	Ag	60–80	Antibacterial activity	Feroze et al., 2020
*Aspergillus niger*	Ag	13.2–646.8	Antifungal effect	Gursoy, 2020
*Acinetobacter baumannii*	Ag	37–168	Antimicrobial and antibiofilm activities	Shaker and Shaaban, 2017
Thermophilic *Bacillus* sp. AZ1	Ag	9–32	Antimicrobial activity	Deljou and Goudarzi, 2016
*Actinomycetes*	Ag	10–20	Antibacterial	Abdeen et al., 2014
*Verticillium* sp.	Ag	25 ± 12	Antimicrobial activity	Mukherjee et al., 2001
*Pseudomonas stutzeri* AG259	Ag	200	Deal with the metal toxicity stress in the environment	Klaus et al., 1999
*Spirulina platensis*	Au	15.60–77.13	Antiviral activity	El-Sheekh et al., 2020
*Sargassum cymosum*	Au	7–20		Costa et al., 2020
*Cladosporium* sp.	Au	5–10	Photodegradation, *in vitro* anticancer activity, and *in vivo* antitumor studies	Munawer et al., 2020
*Morchella esculenta*	Au	16.51	Antimicrobial activity and cytotoxic activity	Acay, 2020
*Micrococcus yunnanensis*	Au	53.8	Antibacterial and anticancer	Jafari et al., 2018
*B. subtilis*	Au	20–25	Catalytic degradation of dye	Srinath et al., 2018
*Shewanella loihica*	Au	2–15	Dye degradation	Ahmed et al., 2018
*Aspergillus* sp.	Au	2.5–6.7	Nitrophenol reduction	Shen et al., 2017
*Streptomyces griseoruber*	Au	5–50	Catalytic activity for the degradation of methylene blue	Ranjitha and Rai, 2017
*Stephanopyxis turris*	Au	10–30		Pytlik et al., 2017
*Galaxaura elongate*	Au	3.85–77	Antibacterial	Abdel-Raouf et al., 2017
*Cystoseira baccata*	Au	8.4	Anticancer	Gonzalez-Ballesteros et al., 2017
*Tetraselmis kochinensis*	Au	5–35		Senapati et al., 2012
*Streptomycetes viridogens* HM10	Au	18–20	Antibacterial activity	Balagurunathan et al., 2011
*Cordyceps militaris*	ZnO	10–15	Photocatalytic degradation of methylene blue dye	Li et al., 2019
*A. niger*	ZnO	53–69	Antibacterial and dye degradation	Kalpana et al., 2018
*Lactobacillus sporogens*	ZnO	145.70	Antimicrobial	Mishra et al., 2013b
*Aeromonas hydrophila*	ZnO	57.7	Antimicrobial activity against *Pseudomonas aeruginosa* and *A. flavus*	Jayaseelan et al., 2012
*Candida albicans*	CdS	50–60	Bactericidal potential against *Salmonella typhi* and *Staphylococcus aureus*	Kumar et al., 2019
Bacteria strains NS2 and NS6	PbS	40–70	Bioremediation without producing toxic chemicals to the environment	Lok et al., 2006
*S. loihica*	Pt	1–10	Dye degradation	Ahmed et al., 2018
*Penicillium chrysogenum*	Pt	5–40	Cytotoxicity	Subramaniyan et al., 2018
*S. loihica*	Pd	1–12	Dye degradation	Ahmed et al., 2018
*S. platensis*	Pd	10–20	Adsorbent	Sayadi et al., 2018
*S. loihica*	Cu	10–16	Antibacterial	Lv et al., 2018
Baker’s yeast	Fe_2_O_3_	2–10	Detection H_2_O_2_ and glucose	Mishra et al., 2015
*Sargassum wightii*	ZrO_2_	18	Antibacterial	Kumaresan et al., 2018
*C. pyrenoidosa*	CdSe QD	4–5	Imatinib sensing	Zhang Z. et al., 2018

*QD, quantum dot.*

### Bacterial-Mediated Synthesis of Nanoparticles

Bacteria that reduce metals are found to be environmentally friendly catalysts for bioremediation as well as material synthesis. In general, through the microbial respiration processes, genus *Shewanella* has been found to synthesize diverse metal oxides (Kim et al., 2018). Electrons can be moved from reduced organic to oxidized inorganic compounds through microbial dissimilatory anaerobic respiration, thus promoting the formation of crystal along with bioremediation processes. It is well documented that the genus *Shewanella* is able to assist the oxidation of organic acids as electron donors and reduction of inorganic metals as electron acceptors (Heidelberg et al., 2002; Harris et al., 2018). Bacterial nanowires and flavins are extracellularly excreted by the genus *Shewanella* by bioreduction process (Marsili et al., 2008; El-Naggar et al., 2010; Beblawy et al., 2018). Few researchers have documented biosynthesis of copper NPs through biosorptive process with dead *Rhodotorula mucilaginosa* biomass. The synthesized NPs were spherical in form and were considered to be a good method for NP synthesis for simultaneous pollutant remediation. Another analysis involving *Clostridium pasteurianum* metallic molybdenum NP synthesis has also been published (Salvadori et al., 2014; Nordmeier et al., 2018). Ag NPs were synthesized by *Shewanella oneidensis* MR-1 with antibacterial activity (Suresh et al., 2010). Another report showed that Cu NPs were synthesized through bioreduction of Cu(II) by *S. oneidensis* MR-1 (Kimber et al., 2018). *Shewanella loihica* PV-4 and *S. oneidensis* MR-1 have the ability to produce Pd NPs with higher catalytic activities (Wang W. et al., 2018; Xiong et al., 2018). *S. oneidensis* MR-1 have properties to utilize toxic soluble tellurite as electron acceptor leading to Te nanorod formation. Also, needle-shaped crystalline Te nanorods were formed both intracellularly and extracellularly (Kim et al., 2012, 2013). Gold- and tellurite-containing nanostructures were biosynthesized in aerobic and anaerobic conditions by crude extracts from *Enterobacter cloacae* MF01 (Contreras et al., 2018). In another study, the investigators explored the formation biogenic selenium nanostructures by gram-negative bacteria under aerobic conditions (Piacenza et al., 2018). Studies have also revealed the importance of different volatile sulfur compounds (VSCs) in the biosynthesis of CdS quantum dots (QDs) by *Pseudomonas fragi* GC01 (Gallardo Benavente et al., 2019). Also, similar studies showed ruthenium and ruthenium-palladium NPs synthesized by *Escherichia coli* cells (Macaskie et al., 2019) and mercury NPs synthesized by *Enterobacter* sp. (Sinha and Khare, 2011).

### Fungus-Mediated Synthesis of Nanoparticles

Fungal NP synthesis is favored over other microbial synthesis methods due to the high resistance of fungal mycelial mesh to higher flow and agitation in bioreactors (Saravanan et al., 2020). Chitin was found to be the key ingredient in the fungal cell wall system that is involved in heavy metal complexation, resulting in synthesis of NPs (Wang L. et al., 2018). Due to several bioactive metabolites, strong aggregation, and increased efficiency, fungi are more resourceful than bacteria in the biosynthesis of NPs (Castro-Longoria et al., 2011; Alghuthaymi et al., 2015; Saravanan et al., 2020). In Au NP biosynthesis, many filamentous fungi have been reported to be capable. This research employed diverse approaches in order to biosynthesize Au NPs. The authors proposed that the NPs could be stabilized by fungal secreted compounds and media materials (Molnar et al., 2018; Guilger-Casagrande and De Lima, 2019). Formulation of silver NPs from *Penicillium chrysogenum* NG85 and *Fusarium chlamydosporum* NG30 was successful. Ag NPs were prepared using cell-free filtrate obtained from the autolyzed biomass. The involvement of enzymes and proteins in the cell-free filtrate was responsible for the formation of NPs in which the enzymes mediated the reduction of silver ions to silver atoms and the stabilization of silver atoms by capping materials was carried out by proteins (Khalil et al., 2019). *Phomopsis liquidambaris* extracellular filtrate isolated from healthy *Salacia chinensis* leaves contains proteins that could reduce and serve as a capping agent, thereby stabilizing the formulation of Ag NPs that demonstrated bactericidal activity against pathogens (Seetharaman et al., 2018). Filtered biomass extract of *Aspergillus tamarii*, *Aspergillus niger*, and *Penicillium ochrochloron* could act as a potential fungal nanofactories for the green and eco-friendly production (Devi and Joshi, 2015). An endophytic fungus *Periconium* sp. isolated from leaves of *Balanites aegyptiaca* mediated the synthesis of zinc NPs (Ganesan et al., 2020). The synthesis of copper oxide NPs from endogenous fungi has been accompanied by a similar method. Copper oxide NPs were synthesized using *Trichoderma asperellum* water extract and copper nitrate solution (Saravanakumar et al., 2019). *P. chrysogenum* provided a particular pigment that facilitated the synthesis of NPs (El-Sayyad et al., 2018).

### Mechanistic Aspect of Metal Nanoparticle Toxicity on Microbes

Nanoparticles have been synthesized from many metals, including gold, silver, copper, nickel, cobalt, zinc, and titanium inside microorganisms. These metal and metal oxide nanomaterials comprise a large segment of the growing nanotechnology market. Increasing use of metallic nanomaterials is likely to result in the release of these particles into the aqueous environments, thus providing a path to enter the food chain and eventually disturbing the ecological balance. Toxicities associated with NPs in microorganisms are mainly related to their nano-size that causes membrane disorganization, generation of reactive oxygen species (ROS), and, in some cases, oxidative DNA damage and release of the toxic ions (Morones et al., 2005; Niazi and Gu, 2009).

The metal NPs cause adsorption on the cell wall leading to depolarization and increase in its permeability, followed by disintegration of membranes and further penetration into the membrane (Thill et al., 2006; McQuillan et al., 2012; Slavin et al., 2017). Inside microbes, these metallic NPs increase significant production of ROS, which target multiple sites simultaneously like conformational changes in protein, peroxidation of the lipids, and DNA damage, thus leading to membrane disintegration and ultimately may cause cell death (Madl et al., 2014; Slavin et al., 2017).

### Purification and Characterization of Monodisperse Nanoparticles

**FIGURE 2:**
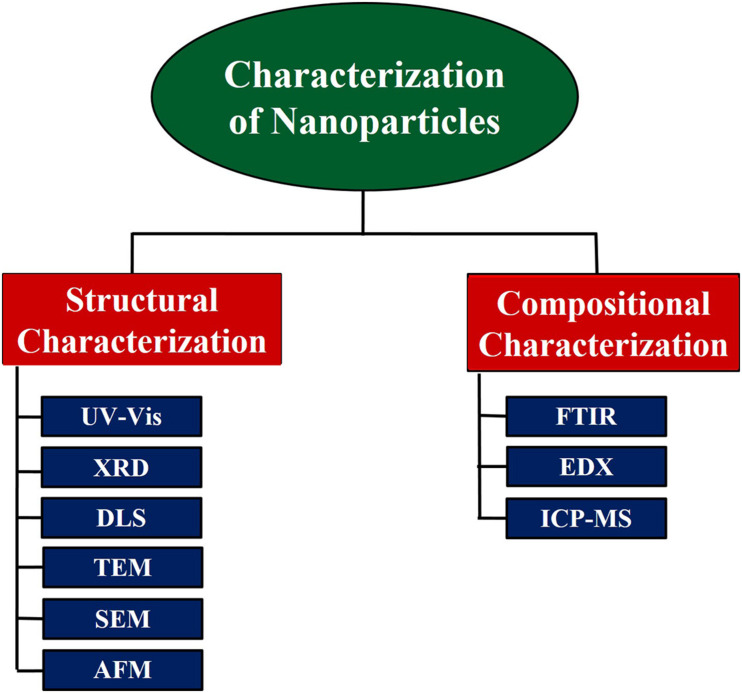
Characterization methods for nanoparticles: several analytical techniques are employed to isolate and characterize monodisperse nanoparticles. UV–Vis, ultraviolet–visible spectroscopy; XRD, X-ray powder diffraction; DLS, dynamic light scattering; TEM, transmission electron microscopy; SEM, scanning electron microscopy; AFM, atomic force microscopy; FTIR, Fourier transform infrared spectroscopy; EDX, energy-dispersive X-ray spectroscopy; ICP-MS, inductively coupled plasma mass spectrometry.

Ensuring monodispersity of NPs is important for biological studies and clinical translation. An important step in understanding the physical properties of NPs is purifying NPs into monodisperse fractions. NPs have been separated by size and form, and certain methods have been created to purify subpopulations of particles after their assembly. Size exclusion chromatography will separate both hard and soft NPs according to their size (Wei and Liu, 1999; Ahmad et al., 2013b; Satzer et al., 2014). Similarly, different density and mass-based methods can separate NPs according to their form (e.g., discoidal from spherical-like NPs) (Johansson et al., 2007; Robertson et al., 2016). There are a variety of techniques that manufacture uniformly appearing nanomaterials with desired properties. The assembly process can yield NPs with several properties simultaneously (Robertson et al., 2016). Characterization methods assess the form, scale, distribution, surface morphology, and surface area of the NPs. Methods to classify NPs are illustrated in Figure 2. The visual observation of color change is critical for NPs. Due to the variation in the surface plasmon resonance (SPR) measured by the NPs, color shift is observed (Zhang et al., 2016). SPR allows the presence of the metal to be monitored by UV–Vis spectroscopy, while X-ray diffraction (XRD) can be used to classify metal NPs. Analysis of XRD data will tell about the composition of materials. Apart from controlling the size of the dust, it is used for particle size determination (Reddy et al., 2016). Dynamic light scattering (DLS) is one of the widely used techniques for the determination of NP size. Hydrodynamic radii are determined using the time it takes light to pass around the particle. In colloidal suspensions, DLS helps ensure calculations are more precise (Kumari et al., 2019). Transmission electron microscopy (TEM) and scanning electron microscopy (SEM) are other useful techniques for the determination of size, shape, morphology, and aggregation of NPs (Ahmad and Khare, 2018). Particle size, agglomeration, and shape can be determined by atomic force microscopy (AFM) topography imaging (Ahmad and Sardar, 2014). Biomolecules responsible for capping NPs can be observed by Fourier transform infra-red (FTIR) spectroscopy (Menon et al., 2017; Sardar et al., 2018). Inductively coupled plasma mass spectrometry (ICP-MS) can analyze NPs qualitatively and quantitatively. Single or combination methods are used to characterize and isolate monodisperse particles.

## Mechanisms Involved in Metal Nanotization

Why do microbes accumulate nanometals? Presence of metals in the environment entails their penetration into cells, disruption of membrane structure, inactivation of enzymes, and production of antimetabolites, which chelate with essential metabolites leading to toxicity or cell death for the microbial cells in the vicinity of the metal particles (Sobolev and Begonia, 2008). This property of metals has also been explored in case of antimicrobial activity against pathogenic strains. As such, microbes try their best to evade the toxicity of the metals by eliminating or reactively changing their properties or accumulating them in forms away for vital organelles. The nanotization of metals by microbes is an important part of geo-cycles and occurs mainly as a stress response resulting in adaptive or defense mechanism for the organism against the metal toxicity. Thus, the microorganisms capable of NP synthesis are often metal resistant in nature, and bacteria genera such as *Pseudomonas* are reported to grow in high metal concentrations (Iravani, 2014; Luo et al., 2015). The interaction of the anionic cell components with the cationic metal ions leads to the formation of metal, metal oxide, or metal sulfide NPs *via* reduction, chelation, or hydrolysis (Bansal et al., 2005). As detoxification measures, the organism can employ enzymatic reduction, dissimilatory oxidation, precipitation, complexation, or transport *via* efflux systems to remediate the metals from the cell (Arshad, 2017). The broad mechanisms of NP synthesis mainly involve either of the pathways involving enzymes, proteins, exopolysaccharides, and electron shuttle quinines (Gahlawat and Choudhury, 2019). Often external parameters such as temperature and pH play a definite role in NP synthesis with regard to properties such as size as well as concentration (Saklani et al., 2012).

Synthesis through microbial routes gives the distinct advantages of control of shape and size, in addition to being a greener alternative to chemical and physical processes. Compared with other biological synthesis methods such as plants, which may result in decrease in monodispersity due the presence of phytochemicals and yield difference due to seasonal variations, microbial means offer a monodisperse NP synthesis methods, which are reproducible in industrial conditions in bioreactors, without batch variations (Ahmad et al., 2021). Additionally, the small size and biocompatibility of biogenic NPs render them good candidates as drug delivery carriers, antimicrobials, anticancer drugs, and diagnostic agents. The disadvantages, though few, mainly refer to the high cost and technology involved in the development and production process of biogenic NPs. Moreover, some NPs may end up in the environment with polluting as well as disease-causing effects (Parveen et al., 2016). Thus, it is important to assess the toxicity and long-term effects of synthesized NPs before using them in any application.

### Enzymatic Action

Enzymes are believed to be the foremost entities involved in the reduction and capping of metals in microbes, through redox reactions occurring in either the intracellular or extracellular space, often acting as the nucleation sites. Broadly, the intracellular route involves metal capture, enzymatic reduction, and capping, while in the extracellular method, secreted or membrane-bound enzymes/proteins are involved. Additionally, shuttle quinones such as anthraquinones, naphthoquinones, and hydroquinones are involved in the process. Mostly NADH-dependent nitrate reductases are reported in fungi to be involved in the reduction process. Fungal species such as *Penicillium brevicompactum*, *Cladosporium cladosporioides*, *Fusarium oxysporum*, *Fusarium semitectum*, and *Fusarium solani* have been reported to use nitrate reductases to produce Ag NPs (Ovais et al., 2018; Meenakshi, 2020). NADH and NADH-dependent reductases in *Pseudomonas aeruginosa* JS-11 were reported for the biogenic reduction of SeO_3_^2–^ to insoluble Se^0^ NPs (Dwivedi et al., 2013). In a cell-free study, glutathione (GSH), NADPH, and GSH reductase have been utilized to quasi-biosynthesize Ag_2_Se QDs with tunable fluorescence (Gu et al., 2012). In *Rhodopseudomonas capsulate*, NADH and NADH-dependent enzymes aided in the conversion of Au ions into Au nanospheres through an electron shuttle mechanism (He et al., 2007). In case of palladium NPs, hydrogenases have been found to play a profound role in reducing Pd(II), with the deposit of Pd(0) NPs depending on the localization of hydrogenases in *Desulfovibrio fructosivorans*. The enzyme serves as the nucleation site, providing electrons to Pd(II) for its reduction (Mikheenko et al., 2008). Hydrogenases have also been reported in the reduction of several other metals such as selenium by *C. pasteurianum*, uranium by *Micrococcus lactyliticus*, and gold by *Shewanella algae* (Iravani, 2014). In case of metal sulfide NPs, the metal and sulfide moieties need to be present in soluble salt form as precursors, and furthermore, sulfide anions and metal cations react with each other to form the metal sulfide NPs, mediated by extracellular or intracellular enzymes. For intracellular reduction, the ions enter the cytoplasm through magnesium or manganese transport chains. For extracellular synthesis, secreted enzymes or those present on the cell membrane are involved (Hosseini and Sarvi, 2015; Fariq et al., 2017). In case of intracellular synthesis, cations are utilized in the capturing metallic ions from the exterior and subsequent reduction inside the cell, followed by accumulation in the cytoplasmic membrane, cell wall, or periplasmic space. Besides the commonly reported reductase, laccase and ligninase have also been reported for the intracellular synthesis route (Ovais et al., 2018).

### Proteins and Peptides in Biosynthesis

In addition to the enzymes discussed in the above section, myriad proteins are also found to responsible for NP formation by microbes. Proteins and peptides are also involved in the capping and stabilization of the formed nanometals. Also, for intracellular entry of metals, transport proteins become essential. Especially in case of magnetic NPs, MTB employ magnetosome membrane proteins for biomineralization. Another protein termed MagA, isolated from the strain *Magnetospirillum* sp. AMB-1, was elemental in biogenic magnetic NP synthesis (Matsunaga and Takeyama, 1998). In another instance, for controlled size and shape, a small acidic protein Mms6 isolated from the same strain was utilized to precipitate uniform CoFe_2_O_4_ nanocrystals *in vitro* (Prozorov et al., 2007). In case of smaller peptides, dipeptides, and tripeptides having polar amino groups have shown mediation of assembly of Au NPs in the presence of HAuCl_4_ as a reducing agent. Gold NP-tripeptide was prepared using a novel tripeptide, harboring a C-terminus tyrosine residue that reduced Au^3+^ to Au NPs. Furthermore, the terminally located free amino group bound Au NPs resulting in stable colloidal Au (Ramesh et al., 2015). In a similar study, yeast strains of *Schizosaccharomyces pombe* and *Candida glabrata* accumulated CdS NPs inside the cell plasma coated with phytochelatins, a peptide known to arrest DNA disruption and cell cycle damage occurring in metal toxicity (Krumov et al., 2007).

### Efflux Pump Systems

As toxic metal ions accumulate around a microbial cell and further enter inside the cell membrane, efflux pumps try to eliminate and excrete them into the extracellular space, thus combating metal toxicity. The presence of efflux pump genes, including a multidrug resistant (MDR) one, is one of the main factors behind a metal-resistant phenotype and has an impact on the nanometal synthesis capacity of the organism. In case of silver, BaeSR, as a two-component signal transduction (TCS) system, is involved in the overexpression of efflux pumps related to metal and antibiotic resistance. Such proteins generally belong to the RND (resistance, nodulation, and cell division) family. Similarly, CusCFBA efflux system has been identified in the *Escherichia coli*, overexpressed in the presence of higher concentration of copper ions (Graves et al., 2015). A lower number of porins such as OmpF or OmpC present on the outer membrane of *E. coli* is also known to be responsible for the efflux of silver ions and hence the resistant phenotype (Salas-Orozco et al., 2019). These efflux pumps are also responsible for antibiotic resistance in bacteria, and one study explored vanillin-coated Au NPs as inhibitors of MexAB-OprM efflux pump components (Arya et al., 2019).

### Interaction With Organelles and Biomolecules

The interaction of metals with cells and subsequent nanosynthesis can be elucidated precisely by understanding how they interact with individual organelles and biomolecules. In fact, instead of whole cells, organelles and subcellular components in *ex vivo* conditions have been explored for their capability to synthesize nanometals with controlled dimensions. In one such instance, circular plasmid DNA molecules close to 4-kb size (acting as the reducing agent) were allowed to complex electrostatically with Ag ions, followed by UV irradiation, leading to the formation of Ag NPs, compared with the non-plasmid control (Liu et al., 2012). Isolated porins from the cell membrane of *Mycobacterium smegmatis*, termed as MspA, were docked onto the self-assembled organic thiosulfates and electrodeposited on Au plates. These self-assembled protein layers were further used to deposit Cu NPs with potential applications in transistors (Woerner et al., 2007). Separate classes of studies have also focused on protein self-assemblies into nanostructures. Genetically engineered *Pichia pastoris* has been used to synthesize protein nanostructures composed of blocks to coat DNA used in gene delivery applications (Hernandez-Garcia et al., 2012).

## Genetic and Molecular Bases of Metal Nanotization

In the presence of elevated concentrations of heavy metals, bacteria usually respond by expressing specific heavy metal resistance genes (MRGs) for NP production. Since these genes are mostly present in plasmids or transposons, they are easily transferred to the neighboring microbial communities. Several research groups have studied these genes to elucidate the mechanisms behind nanotization. A group worked on estimating the amount of metals bioavailable for the microbial community using quantitative PCR and found that the gene *czcA* (a Cd/Zn/Co efflux pump) was responsible for Cd/Zn/Co bioavailability in microbes (Roosa et al., 2014). A study by yang and their group showed that some of the MRG such as *pcoA*, *merA*, *silC*, and *arsA* genes were present in higher frequencies in MDR *bla*_NDM–1_^–^ and *bla*_CTX–M–15_^–^ Enterobacteriaceae isolates (Yang et al., 2018). In Northern China near a copper tailing dam area, the following genes were found at the remedial site: copper resistance genes (*copA*, *copB*, *pcoA*, *pcoC*, and *pcoD*), other MRG (*czcA*, *czcC*, and *czcD*), arsenic resistance genes (*arsB* and *arsC*), *nccA* (for nickel), *pbrT* (for lead), and *chrB* (for chromium) (Chen et al., 2019). Some of the genes are present in operons such as s*ilG* gene in the *sil* operon for silver sequestration (Randall et al., 2015). A study has shown that *arsRBCC* operon and *arsC* gene in *Desulfovibrio desulfuricans* G20 helped in regulating arsenic in the microbial environment (Li and Krumholz, 2007). Studies have also found upregulated levels of efflux complexes in the presence of excess heavy metals, such as resistance-nodulation-cell division (RND) transporters, the P-type ATPases, efflux complexes made from membrane fusion protein (MFP) family, or the outer membrane factor (OMF) protein family.

The understanding of the genetics of microbe-mediated NP synthesis has been interestingly used to design genetically engineered microbes with metal-resistant phenotypes, which find applications in NP synthesis as well as remediating the metal-contaminated environment. Such an example was observed in *Escherichia coli* strains expressing PC synthetase of *Schizosaccharomyces pombe*, used for the synthesis of semiconductor CdS nanocrystals. The phytochelatins produced by the action of PC synthase act as a nucleation site for the nanocrystals and stabilize them against aggregation (Kang et al., 2008). Thus, several diverse gene families are reported to be involved in nanotization of metals by microbial strains.

## Applications of Microbial Nanoparticles

Owing to their nanoscale sizes leading to an increase in the surface/volume ratio, NPs find applications in myriad industrial, environmental, and biomedical applications. TiO_2_ NPs from several microbial species such as *Micrococcus lylae*, *Cellulosimicrobium* sp., *Micrococcus aloeverae*, and *Chlorella pyrenoidosa* have been shown to show dye-degradation capability to remediate polluted waste waters (Fulekar et al., 2018). Similarly, Au NPs from *Streptomyces griseoruber* and ZnO NPs from *Cordyceps* have been specifically explored to degrade methylene blue dye *via* catalysis (Ranjitha and Rai, 2017; Li et al., 2019). Toxic compound remediation is also a major environmental application of biogenic NPs where PbS and Au NPs from bacterial and fungal strains have shown promise (Lok et al., 2006; Shen et al., 2017). In electronics, TiO_2_ NPs from *Bacillus mycoides* have been applied in the construction of green solar cells (Ordenes-Aenishanslins et al., 2014). Closer dimensions to biomolecules also render these biogenic NPs good candidates for biomedical applications. A major portion of microbial NPs such as Ag, Ag, Se, and Te NPs find applications as antibacterial, antifungal, antiviral, and antibiofilm agents (Abdeen et al., 2014; Zonaro et al., 2015; El-Sheekh et al., 2020; Gursoy, 2020). Biogenic NPs display good penetration across membranes, and blood–tissue and blood–brain barriers, thus finding applications as anticancer agents and drug delivery vehicles (Gonzalez-Ballesteros et al., 2017). An interesting application had utilized bacterial magnetosomes loaded with doxorubicin and tested on H22 tumor-bearing mice; they displayed higher tumor toxicity than only doxorubicin (Sun et al., 2009). These magnetosomes are also recognized as better MRI contrast agents with higher relaxivity than the conventional ones (Zhang Y. et al., 2018). In biosensing, CdSe QDs from *C. pyrenoidosa* have been exploited for imatinib sensing, while Fe_2_O_3_ NPs have been used to detect H_2_O_2_ and glucose (Mishra et al., 2016; Zhang Z. et al., 2018). Thus, it is evident that the applications of microbial NPs are diverse and multifaceted, holding great promise in several fields of research.

## Conclusion and Future Perspectives

Several bacterial, viral, algal, fungal, and yeast species are known to trap metals *in situ* and convert them to elemental NP forms, while remediating their immediate environment in the process. This feature has been further exploited in industrial, environmental, and biomedical applications. However, most metals are toxic to microbial cells; thus, it is widely reported that the synthesis of metals into their elemental nano-forms results as a defense mechanism or stress response for the organism to eliminate, segregate from essential organelles, or reactively change the harmful nature of the metals. Mechanisms such as enzymatic reactions, precipitation, complexation, binding to peptides, and efflux pumps are involved in this process, which act independently or simultaneously for the metal remediation by the cell, resulting in a metal-resistant phenotype, with microbes harboring specific genes for this property. Even though a number of mechanisms have been reported for biogenic nanosynthesis, it is important to extend our studies to other reducing enzymes, catalytic proteins, and stabilizers along with their tandem action in the cell. The role of different classes of enzymes needs to be studied in detail. The precise understanding of pathways and mechanisms involved in the biogenic synthesis allows researchers to modulate existing microbes and engineer metabolic pathways for NP synthesis with controlled size and shapes for varied applications.

## Author Contributions

SG, RA, and KB conceptualized and prepared the manuscript. MFA and SR helped and addressing the review comments with inputs which were further included in the revised manuscript. All authors have critically reviewed the manuscript.

## Conflict of Interest

The authors declare that the research was conducted in the absence of any commercial or financial relationships that could be construed as a potential conflict of interest.
